# Anterior interhemispheric vs. pterional approach in the microsurgical management of anterior communicating artery aneurysms: a comparative analysis employing a novel multidimensional matching-tool

**DOI:** 10.1007/s10143-024-02592-w

**Published:** 2024-07-29

**Authors:** Vanessa M. Swiatek, Amir Amini, Lena Spitz, Ali Rashidi, Claudia A. Dumitru, Klaus-Peter Stein, Sylvia Saalfeld, I. Erol Sandalcioglu, Belal Neyazi

**Affiliations:** 1 Department of Neurosurgery, Otto-Von-Guericke University, Leipziger Straße 44, 39120 Magdeburg, Saxony Anhalt Germany; 2 Department of Simulation and Graphics, Otto-Von-Guericke University, Universitätsplatz 2, 39106 Magdeburg, Saxony Anhalt Germany; 3 Research Campus STIMULATE, Otto-Hahn-Straße 2, 39106 Magdeburg, Saxony Anhalt Germany; 4 Department of Medical Informatics, University Hospital Schleswig-Holstein Campus Kiel, Arnold-Heller-Straße 3, 24105 Kiel, Schleswig-Holstein Germany

**Keywords:** Anterior communicating artery aneurysm, Interhemispheric approach, Multidimensional matching, Pterional approach

## Abstract

The surgical management of anterior communicating artery aneurysms (AcomA) is challenging due to their deep midline position and proximity to complex skull base anatomy. This study compares the pterional craniotomy with the interhemispheric approach based on the specific aneurysm angulation. A total of 129 AcomA cases were analyzed, with 50 undergoing microsurgical clipping via either the pterional or interhemispheric approach. All selected cases had computed tomography-angiography with sagittal imaging slices and 2D-angiography. Using an interactive tool, 14 cases treated via the interhemispheric approach were matched with 14 cases approached pterionally based on clinical and morphological parameters, emphasizing intracranial aneurysm (IA) dome angulation relative to the frontal skull base. Outcomes included IA occlusion, temporary clipping incidence, intraoperative rupture, postoperative strokes, hemorrhages, hydrocephalus, vasospasm, and patient functionality. Matched cohorts had consistent demographics. Both approaches resulted in similar IA occlusion rates, but the interhemispheric approach led to improved clinical outcomes, measured by the modified Rankin Scale. It also had a lower incidence of hydrocephalus and reduced need for permanent ventriculoperitoneal shunt placement. Vasospasms and postoperative infarction rates were comparable between the groups. Our findings suggest potential advantages of the interhemispheric approach in managing AcomA, depending on aneurysm angulation. Despite a small sample size, the results highlight the importance of customized surgical decision-making based on the unique traits of each aneurysm and the surgeon's expertise.

**Supplementary Information:**

The online version contains supplementary material available at 10.1007/s10143-024-02592-w.

## Introduction

Intracranial aneurysms (IA) of the anterior communicating artery (Acom) represent a challenge for neurosurgeons due to their unique location and the potential risks of treatment [14]. Acom aneurysms (AcomA) are frequent and pose a high risk of rupture compared to other types of IA [2, 3, 10, 14, 18, 23, 24, 31]. The microsurgical treatment of AcomA can be challenging due to the intricate anatomy of the area and proximity to vital neuronal structures. AcomA are nestled deep within the midline where they sit at the confluence of several blood vessels including a close anatomical relation to the recurrent artery of Heubner and to several perforating arteries [14, 19, 24]. The bilateral anterograde arterial supply of the Acom significantly amplifies the surgical complexity, as surgeons need to meticulously identify and preserve multiple arteries throughout the dissection. Therefore, surgery requires careful execution to avoid intraoperative IA rupture or damage of the surrounding structures, while also ensuring adequate blood flow to prevent postoperative stroke [14, 24].

Over time, efforts have been made to classify AcomA and its complex based on factors such as aneurysm morphology, rupture status, and position. The angulation of the aneurysm is of particular importance [6, 14, 22, 24]. Various classifications consider the AcomA direction relative to cerebral landmarks or the skull base, affecting surgical approach choices. These highlight the need for detailed preoperative evaluation of AcomA's unique anatomy [14, 28]. The pterional approach (PA), established by Yaşargil in the 1970s, is a standard method for accessing the AcomA complex, known for its effective aneurysm clipping and minimal complications [24, 28, 29]. Conversely, the anterior interhemispheric approach (AIA), developed by Walter E. Dandy in the 1930s and favored in Japan, provides direct access to midline aneurysms, optimizing visualization while preserving adjacent brain structures [4, 6, 16, 24, 30].

The choice of surgical approach often reflects the practices and education at a medical institution. Comparing these approaches based on anatomical and microsurgical considerations could lead to improved personalized treatments. The effectiveness of different surgical strategies for managing AcomA critically depends on the accuracy of patient matching. However, the development of comprehensive multidimensional matching parameters to facilitate the comparison of treatment approaches is challenging and not yet fully realized. Our study employs an interactive visual exploration tool to facilitate a detailed comparative analysis of the AIA and the PA for AcomA treatment.

## Materials and methods

To conduct this study, we examined a previously collected database of 129 patients with AcomA treated at the Department of Neurosurgery in KRH Hospital Nordstadt, Hanover. We conducted a retrospective analysis and applied the following inclusion criteria:

1. Localization at the Acom.
2. Surgical treatment via microsurgical clipping of the AcomA through either a PA or AIA (Fig. 1).

**Fig. 1:**
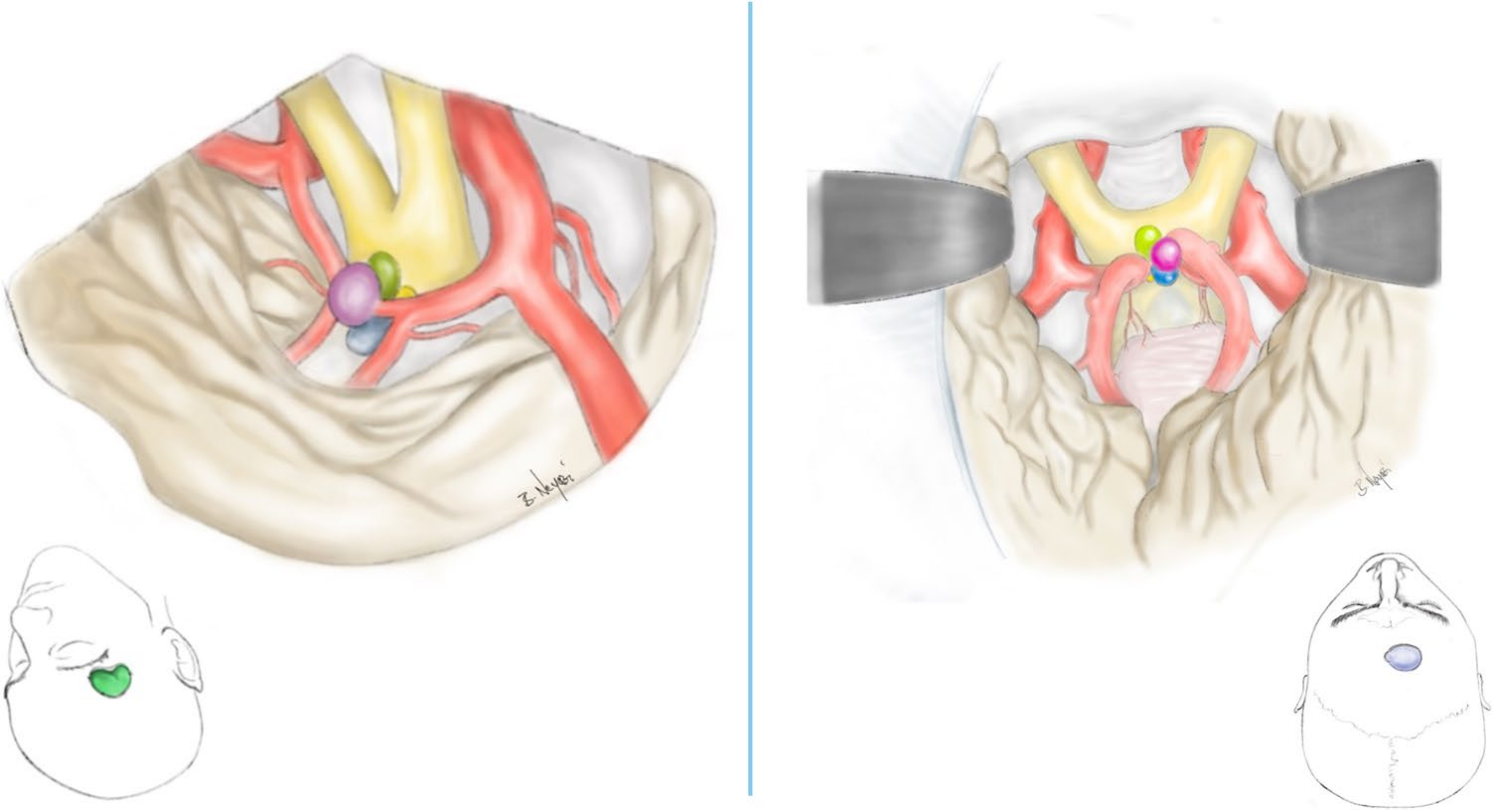
This figure illustrates the surgical approaches utilized, showing both the craniotomy and the microsurgical perspective of the aneurysm and potential angulations. The color coding is consistent with the angle categories shown in Fig. 3, with the pterional craniotomy highlighted in green and the anterior interhemispheric craniotomy in blue
3. Availability of computed tomography-angiography (CTA) with sagittal imaging slices and two-dimensional digital subtraction angiography (2D-DSA).

**Fig. 2:**
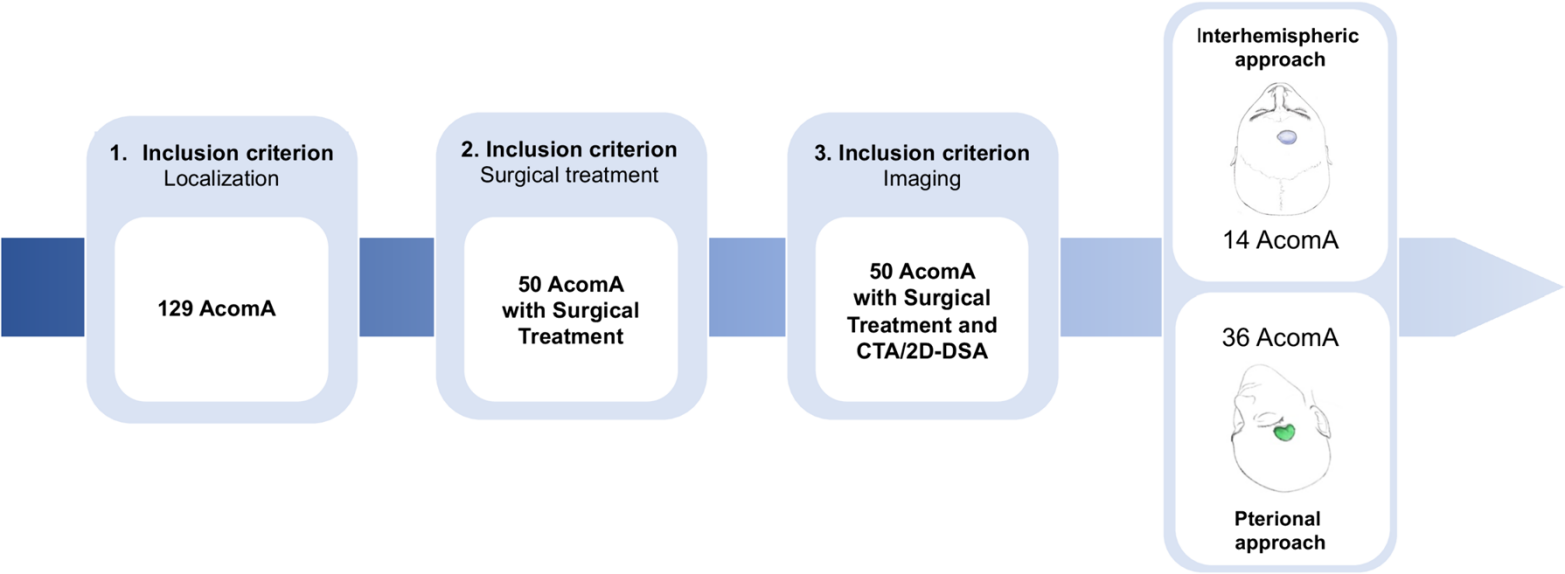
Flow chart illustrating the determination of the study-cohort according to the inclusion criteria and the final cohort matching (AcomA; Anterior communicating artery aneurysm)

All patients underwent microsurgical clipping of the AcomA between 2009 and 2018 (AIA from 2009–2017; PA 2013–2018). Out of the 129 AcomA cases presented to our clinic, only 50 were treated surgically, with the remaining cases managed through endovascular approaches or not treated at all. Given that PA is the standard surgical approach in our clinic, the majority of these cases were treated via PA, with only 14 cases managed via AIA. The workflow leading to the comparison of 14 cases for each method is detailed in Fig. 2.

Once the cohort was defined based on the aforementioned criteria, a matching process was performed to pair all patients who underwent AIA for AcomA clipping with those who underwent PA for AcomA clipping, taking into account similar clinical and morphological parameters (Fig. 2).

### Data acquisition

A thorough review of each patient’s medical record, including patient history, medication registries, and diagnostic imaging was conducted. We focused on cardiovascular diseases, risk factors, and significant conditions like malignant neoplasms or autoimmune disorders requiring immunosuppressive therapy. This allowed us to establish a reliable dataset for identification of appropriate matching criteria. We also extensively analyzed the progression and management of IA, leveraging diagnostic imaging, aneurysm-specific risk factors, and clinical outcomes post-treatment or rupture. Special attention was given to surgical procedure parameters, detailed in Table 1.

**Table 1:** List of examined clinical parameters and their definitions [1, 7–9, 11–13, 15, 17, 20, 27]

Parameter category	Parameters/Definitions
Epidemiological data	Age (defined as age at surgery)
	Gender (defined as biological gender)
Medical history	Hypertension (defined as documented diagnosis or intake of antihypertensive medication)
	Diabetes mellitus (defined as documented diagnosis of type 1 or 2 diabetes or intake of oral antidiabetics or insulin)
	Hyperlipidemia (defined as documented diagnosis or intakt of medication lowering the lipid or cholesterol levels in the blood)
	Peripheral arterial disease (defined as documented diagnosis or imaging finding)
	Heart disease (defined as documented diagnosis of myocardial infarction, coronary artery disease, cardia arrhytmia or other heart diseases)
	Ischaemic stroke (defined as documented diagnosis or imaging finding at admission)
	Thrombosis (defined as documented diagnosis)
	Malignant tumor disease (defined as documented diagnosis regardless of affected organ)
	Autoimmune disease (defined as documented diagnosis with need of immunosuppressive therapy)
	Obesity (defined as a documented body mass index of > 30 kg/m^2^)
	Nicotine abuse (defined as ex-nicotine abuse or continued nicotine abuse)
	Alcohol abuse (defined ot consumption of > 50 g of alcohol per week)
	Contraceptive use and intake of hormone replacement products (at time of diagnosis, extracted from medical records or medication plans)
CT-imaging parameters	Type of bleeding, shifting of the midline, intraventricular hemorrhage, hydrocephalus and ischaemia (all assed in the first CT-scan after SAH)
Aneurysm-related parameters	Rupturestatus (defined by assessment of intraoperative findings, imaging findings and bleeding patterns in CT)
	Multiplicity (defined as > / = 2 intracranial aneurysms)
	Aneurysm localization (defined by assessment of angiography)
Clinical scores	Glasgow coma scale at admission and discharge (defined by neurological examination at admission and discharge)
	Modified rankin scale at discharge (defined by neurological examination at discharge)
	Hunt and Hess grade, WFNS score and Fisher grade at admission (defined by neurological examination at admission and imaging findings)
Treatment-related parameters	Previous and current treatment and treatment-modality (extracted from medical records)
Surgery-related parameters	Number of clips used (extracted from the operation report)
	Type of craniotomy (extracted from the operation report)
	Occurence of intraoperative rupture (extracted from the operation report)
	Need for and duration of intraoperative temporary clipping (extracted from the operation report)
	Use of intraoperative indocyanine green angiography and/or microvascular doppler sonography (extracted from the operation report)
	Need for primary or secondary decompressive hemicraniectomy (extracted from the operation report)
	Occurence and type of postoperative stroke or haemorrhage (extracted from the postoperative CT-scans)
	Degree of occlusion of the aneurysm and need for revision in partially/unoccluded aneurysms (extracted from the postoperative angiography and medical records)
Complication-related parameters	Hydrocephalus, placement of an external ventricular drainage or ventriculoperitoneal shunt (extracted from medical records)
	Vasospasm, method of vasospasm-detection and treatment via endovascular spasmolysis (extracted from medical records)
Follow-up data	Time of follow-up, perfusion of the aneurysm and modified rankin scale at follow-up (extracted from medical records)

### Morphological analysis

The morphological analysis was conducted based on 2D-DSA datasets of the IAs. The parameter definition was adopted from the publication by Dhar et al. and applied to our patient cohort (Table 2). Only the Frontal Base-Dome-Angle (FDA) was measured using sagittal CTA datasets, and its precise definition and explanation are provided in the subsequent section.

**Table 2:** Definition of the 11 manually measured morphological parameters and two visually assessed parameters [5, 21]

Morphological parameters	Definition
Width (W)	Maximum width of the aneurysm
Size (H)	Maximum perpendicular distance of the dome from the neck plane
Neck (D)	Twice the average distance from the neck centroid to the edge of the neck
Parentvessel diameter 1 (D_1_)	Vessel diameter at the proximal neck
Parentvessel diameter 2 (D_2_)	Vessel diameter 1.5 × D_1_ upstream
Maximum height (H_max_)	Maximum distance of the dome from the middle of the neck
Size Ratio (SR)	Ratio of the maximum aneurysm height to the average vessel diameter
Aspect ratio (AR)	Ratio of the maximum perpendicular height to the average neck diameter
Vessel angle	Angle between the inlet vessel centerline and the neck plane
Inclination angle	Angle of inclination between the IA and its neck plane
Frontal base-dome-angle	Angle between the maximum height of the aneurysm and the frontal skull base in sagittal CT-scans
Appearance of aneurysm bleps	Secondary outpouching of the aneurysm
Aneurysm shape	Visually assessed regular or irregular structure of the aneurysm wall

### Definition and measurement of the FDA

The choice between a PA or AIA for surgical intervention often depends on the surgeon's preference and experience, with various modifications documented in technical notes and studies. Despite discussions on both techniques, comparative analyses of postoperative outcomes for high-positioned AcomA are lacking. Key factors in selecting the surgical approach include clinical parameters (e.g., rupture status, patient's condition, age) and morphological features (e.g., aneurysm size, neck configuration, shape), with the aneurysm's angulation relative to the frontal base being crucial yet underrepresented.

**Fig. 3:**
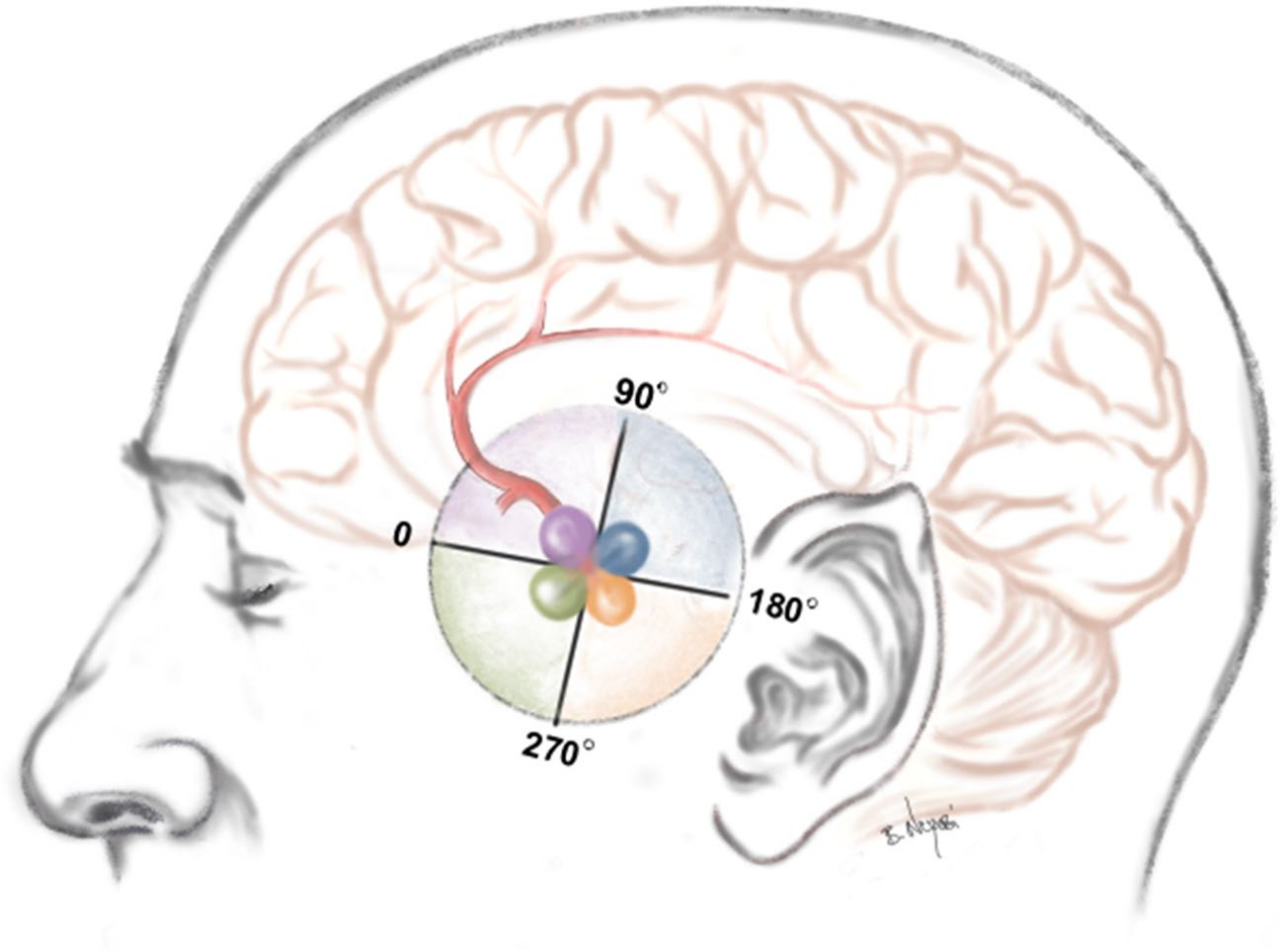
Fig. 3 presents a schematic depiction of the FDA relative to the frontal base. The angles are classified into four categories: category 1 (0–90°) depicted in purple, category 2 (90–180°) in blue, category 3 (180–270°) in orange, and category 4 (270–360°) in green

To address this, we introduced a classification based on the angulation of AcomA in sagittal CT scans, defining a baseline parallel to the frontal base. The angle between this baseline and the aneurysm dome determines the category, with 0° starting ventral to the aneurysm and categories spanning a 360° circular path. Measurements are made on slices best showing the IA height and neck, dividing the frontal base orientation into four quadrants: 0–90° (category 1), 90.1–180° (category 2), 180.1–270° (category 3), and 270.1–360° (category 4) (Fig. 3).

### Matching criteria and cohort matching

To neutralize the influence of significant risk factors on the outcomes, systematic pairing was performed. This strategy was designed to enable a more equitable comparison between the AIA and PA for AcomA surgery, matching the most closely related pterionally treated AcomA to each interhemispherically treated case. Consequently, we matched 14 AcomA treated via AIA against 36 treated via PA, ensuring pairs were within the same FDA category.

One exceptional case with a FDA category 3 was included in the cohort of category 4 AcomA for matching purposes. For the matching of unruptured AcomA, the following parameters were taken into account: gender, age at the time of surgery, aneurysm shape, and aneurysm size. For the matching of ruptured AcomA, the following parameters were considered: gender, age at the time of operation, Hunt and Hess score, Fisher grade, aneurysm shape, and aneurysm size.

Given the considerable number of parameters involved in the matching process, an interactive visual exploration tool developed by Spitz et al., designed for case-based reasoning of IA, was employed [25, 26].

**Fig. 4:**
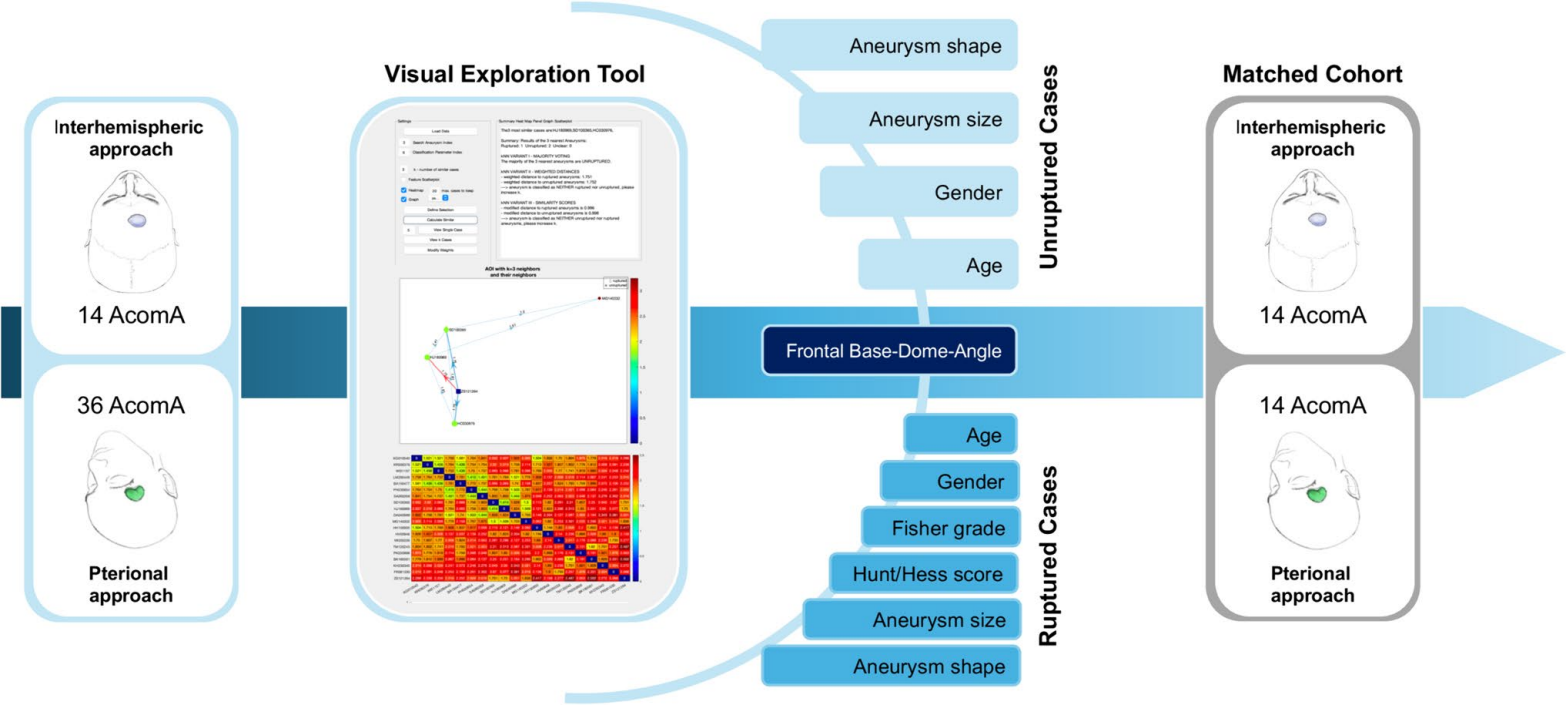
Schematic representation of the matching process following cohort identification. The figure illustrates how 14 through AIA operated AcomA were matched with 36 pterionally approached AcomA using the Visual Exploration Tool. To facilitate the matching process, we carefully selected specific parameters that could potentially influence the postoperative outcome for both ruptured and unruptured cases. The final matched cohort consisted of the 14 AcomA operated via the AIA and the 14 best-matching pterionally approached AcomA

Information on the earlier mentioned parameters was uploaded into the interactive visual exploration tool utilized in our study (Fig. 4). In our methodology, the AcomA treated through an AIA, referred to as the "aneurysm of interest" (AOI), was sequentially replaced with the next one in line following each successful matching. Subsequently, the matching process was initiated again using the updated set of IAs.

To perform IA matching, we utilized case-based reasoning as a method to find the most similar cases treated via a PA to the selected AcomA treated via an AIA, referred to as the AOI. The tool used a method called k-nearest neighbor-based (k-NN) classification to assess the similarity between cases. Specifically, we compared the AOI (AcomA treated via an AIA) to a reference database comprising AcomA treated via a PA. To determine the most similar AcomA treated via the PA to the AOI, we considered the k cases that were closest in similarity (in our case, we set k to three due to the small sample size).

To enable fair comparison, the values of each feature were normalized using the Z-score standardization. The dissimilarity between two IAs, x and q, was calculated using the following formula:

$$\text{dist}(\text{x},\text{ q})=\surd (\sum \text{l}=1\text{N}(\text{fwl}*(\text{xl}-\text{ql})^2))$$

Here, N represents the number of features, xl denotes the value of the l-th feature of IAs x, and fwl represents the weight assigned to the feature. By default, all feature weights were set to 1.

In our study, we used three variants of the k-NN-based classifier. The first variant was a simple k-NN classifier that calculated the dissimilarity between the AOI and all IA in the database. It then selected the k nearest IA, which in our case were three, to determine the nearest neighbors. In the ordinary k-NN classifier, each nearest neighbor had an equal impact on the classification, irrespective of its actual distance to the AOI. To address this, the second variant incorporated the actual distances as weights by assigning a weight to each near IA inversely proportional to the distance. The third variant of the k-NN-based classifier followed a similar approach but included an additional step of normalizing all distances using min–max scaling into the range of [0, 1].

To facilitate interactive exploration and analysis of the data, Spitz et al. developed a visual analytics framework. This framework integrated various visualization techniques, including a summary panel, a directed graph panel, and an interactive heat map. These visualizations allowed users to examine and compare the features of different IA and identify the most similar comparative AcomA (Fig. 4).

**Fig. 5:**
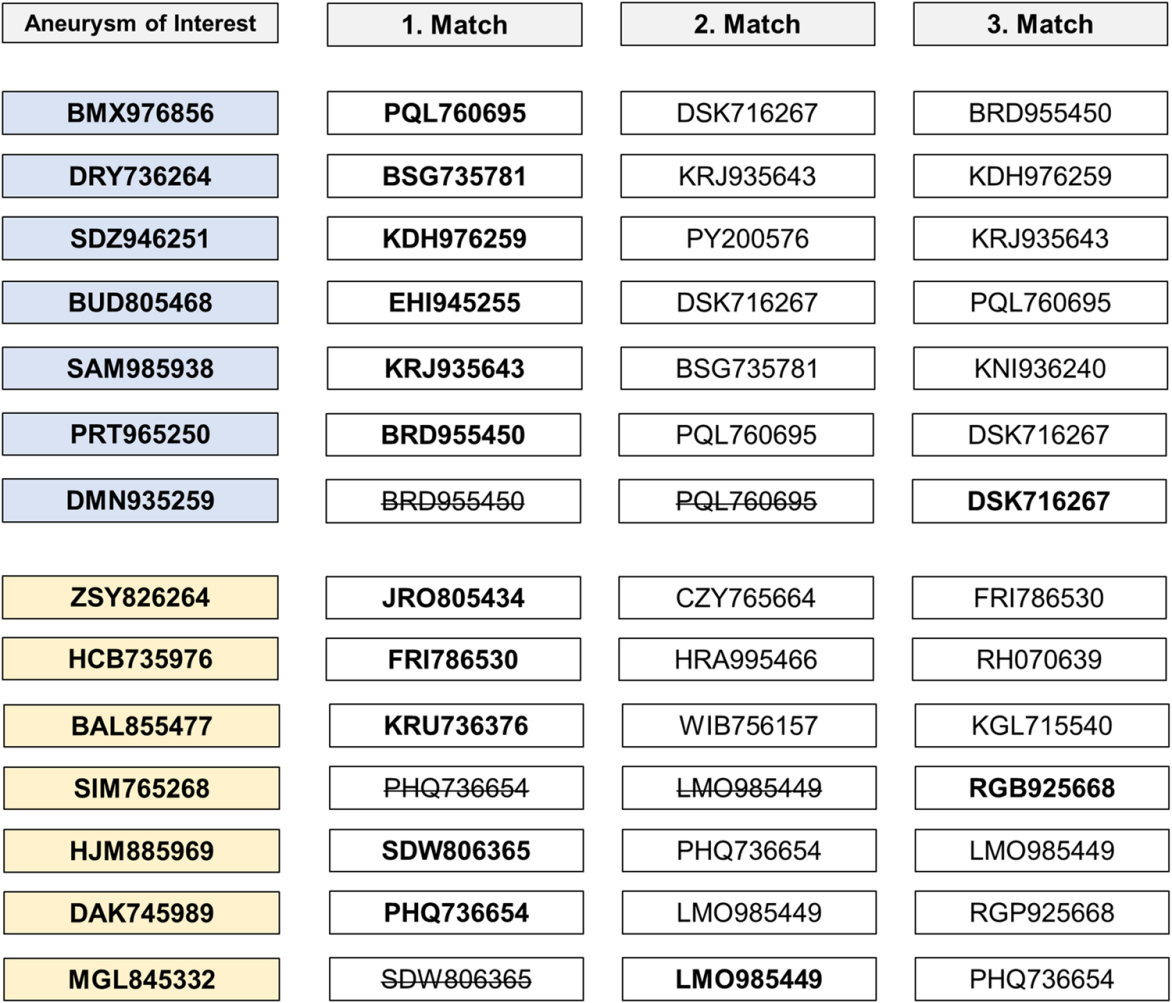
In the matching process, situations arose where an already matched PA treated AcomA was selected as the most similar in additional cases. Among the seven unruptured cases (highlighted in blue), one pterional approached AcomA exhibited the closest similarity to two different interhemispheric approached AcomA. Similarly, among the seven ruptured cases (highlighted in yellow), two cases involved pterional approached AcomA identified as the most similar to two different interhemispheric approached AcomA. To resolve these cases, the second and third matches were evaluated by two independent neurosurgeons to determine the final match. No disagreements occurred between the investigators during this process

During the matching process, we identified the three most similar AcomA treated via a PA to the interhemispherically approached AcomA. Among the seven unruptured cases, one pterionallly approached AcomA was found to be the most similar to two different interhemispherically approached AcomA. Similarly, among the seven ruptured cases, in two cases the pterional approached AcomA were identified as the most similar to two different interhemispherically approached AcomA. In these cases, the second and third matches were independently assessed by two neurosurgeons to determine the final match (Fig. 5). Notably, there were no disagreements between the investigators throughout this process. Consequently, a total of 14 AcomA treated via an AIA were statistically compared with a matched cohort of 14 AcomA treated via a PA.

### Statistical analysis

To analyze and compare the outcomes of the AIA and PA for AcomA surgery, primary and secondary parameters were chosen to measure the effectiveness of surgical therapy. The primary parameters were the degree of aneurysm occlusion (classified as complete or incomplete) and the patients' functional outcomes after surgery, assessed using the modified Rankin Scale (mRS) at discharge. Secondary outcome variables were also examined to evaluate the success of the surgery, including the incidence of temporary clipping, intraoperative rupture, and postoperative strokes or hemorrhages. Additional complications like hydrocephalus and vasospasm were also considered. Statistical analyses were conducted using IBM SPSS Statistics 29. For numerical variables, Chi-square tests were used, with Fisher's Exact Test substituting when any expected cell frequency was below 5. Ordinal or metric variables were first tested for distribution normality using the Kolmogorov–Smirnov test, followed by Levene's test for variance homogeneity. Non-normally distributed data were analyzed with the Mann–Whitney U test, while normally distributed data underwent a t-test.

## Results

### Cohort overview

The cohort of AcomA patients who underwent surgery via an AIA included two males and twelve females, with an average age of 53.1 years. The cohort of AcomA patients treated via a PA consisted of two males and twelve females, with an average age of 56 years. The proportion of ruptured IAs was identical in both cohorts. The average IA size was 10 mm for the cohort treated interhemispherically and 6.3 mm for the cohort treated pterionally.

**Fig. 6:**
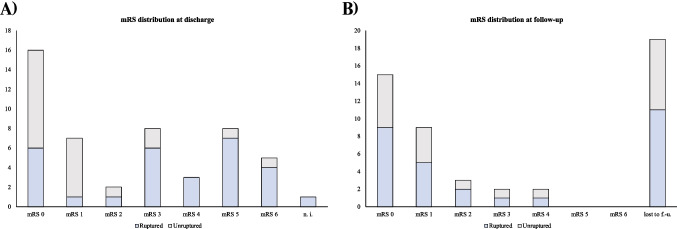
Ruptured cases are indicated in blue, while unruptured cases are depicted in grey. The x-axis represents the number of cases. Panel (**A**) illustrates the distribution of mRS scores at discharge for surgically treated AcomA, and Panel (**B**) shows the distribution of mRS scores at follow-up. (n.i.; no information; lost to f.-u.; lost to follow-up)

The surgical outcomes for the entire cohort of 50 AcomA cases treated surgically are depicted in Fig. 6, detailing the distribution of mRS scores at discharge and follow-up. Information on the mRS at discharge is available for 49 out of 50 patients, while follow-up mRS data is available for 31 patients. The follow-up duration ranged from one to 24 months, with an average of 9.6 months. Among the 19 patients who did not have follow-up data, five had died during their hospital stay. At discharge, four patients had an mRS of five, six had an mRS of zero, two had an mRS of one, and two had an mRS of three. This pattern suggests that patients with very good outcomes may not return for follow-up, whereas those with very poor outcomes may be unable to do so.

During the follow-up period, common post-surgical symptoms such as occasional headaches and local wound discomfort were observed. Typical post-subarachnoid hemorrhage (SAH) symptoms, including headaches, concentration disturbances, and in some cases, motor disturbances and bedridden status due to infarctions, were also noted.

Regarding cranial nerve affections, one patient in the AIA cohort experienced visual field defects due to optic nerve involvement, and one patient in the PA cohort exhibited oculomotor palsy. The visual field defect in the AIA cohort occurred in a patient with an incidental aneurysm, while the oculomotor palsy in the PA cohort was observed in a patient who had suffered SAH. Concerning olfaction, no significant abnormalities were detected among our cohort of 50 surgically treated AcomA patients.

According to our standard protocol, patients with occluded singular IA confirmed by DSA and no further symptoms are not required to follow up regularly in our clinic, but only if symptoms occur.

Detailed data for individual cases of the matched cohort are available for review in the Supplemental Material. Several clinically important aspects, particularly the placement and indication for external ventricular drains (EVD), a crucial component of aneurysm treatment, were not fully addressed in the main results and require further mention.

In the matched cohort, EVD were placed in four patients undergoing the AIA and nine patients undergoing the PA. All four AIA patients and six patients with PA had SAH, while the remaining three patients operated via PA were treated for incidental aneurysms. Our analysis primarily focused on the rate of ventriculoperitoneal shunt dependency post-surgery rather than the placement of EVD. In the majority of SAH cases, the decision to place an EVD was made prior to the selection of the surgical approach for the AcomA, indicating that this factor was not influenced by the surgical approach. However, the varying rates of long-term ventriculoperitoneal shunt dependency might reflect different surgical strategies.

SAH patients in the analyzed cohort had their EVD placed before surgery, with right frontal EVD placement being the standard procedure. There was only one instance of left frontal EVD placement, and no lumbar drains were utilized. For the three patients with incidental aneurysms who required EVD placement, this was due to intraoperative complications, and the EVD were placed either perioperatively or postoperatively.

The matched cohort of 14 interhemispherically treated AcomA and 14 pterionally treated AcomA was subsequently compared in terms of numerous other clinical patient- and aneurysm-specific parameters following the matching process. Data are presented in absolute numbers and percentages in Table 3. The effectiveness of the matching process was substantiated through a statistical analysis, which did not reveal any significant differences between the cohorts.

**Table 3:** This figure delineates a comparison of epidemiological data, pre-existing conditions, rupture status, aneurysm multiplicity, aneurysm size and irregularity, along with median Hunt and Hess scores and Fisher grades for ruptured AcomA across the matched cohort

	Interhemispheric approach (*n* = 14)	Pterional approach (*n* = 14)	Statistical analysis
Gender	2 male; 12 female	2 male; 12 female	*p* = 0.702****
Age at surgery (mean)	53.1 years	56 years	*p* = 0.511***
Hypertension	5 (35.7%)	6 (42.9%)	*p* = 0.5****
Diabetes mellitus	0 (0%)	0 (0%)	—
Peripheral arterial disease	1 (7%)	0 (0%)	*p* = 0.5****
Heart disease	3 (21.4%)	2 (14.3%)	*p* = 0.5****
Ischaemic stroke	3 (21.4%)	0 (0%)	*p* = 0.111****
Thrombosis	0 (0%)	0 (0%)	—
Obesity	1 (7%)	1 (7%)	*p* = 0.759****
Nicotine abuse	5 (35.7%)	5 (35.7%)	*p* = 0.653****
Alcohol abuse	0 (0%)	0 (0%)	—
Ruptured aneurysms	7 (50%)	7 (50%)	*p* = 0.647****
Multiple aneurysms	5 (35.7%)	4 (28.6%)	*p* = 0.5****
Aneurysm size (mean)	10 mm	6.3 mm	*p* = 0.802***
Aneurysm irregularity	3 (21.4%)	6 (42.5%)	*p* = 0.21****
Hunt and Hess score (median)	1	2	*p* = 0.632**
Fisher grade (median)	3	2	*p* = 0.254**

Included are tests to evaluate differences between cohorts using the Mann–Whitney-U-test(**), t-test (***)and Exact-Fisher-test(****)

### Outcome analysis

For outcome analysis and comparison of the AIA and PA for AcomA clipping, primary and secondary parameters were selected to evaluate the efficacy of surgical therapy. The primary parameters included the degree of aneurysm occlusion (complete/incomplete) and the functional outcome of patients after surgery, as measured by mRS at discharge. Additionally, secondary outcome variables were examined to assess surgical success, encompassing the incidence of temporary clipping, intraoperative rupture, postoperative occurrences of strokes or hemorrhages and complications like hydrocephalus and vasospasm.

**Fig. 7:**
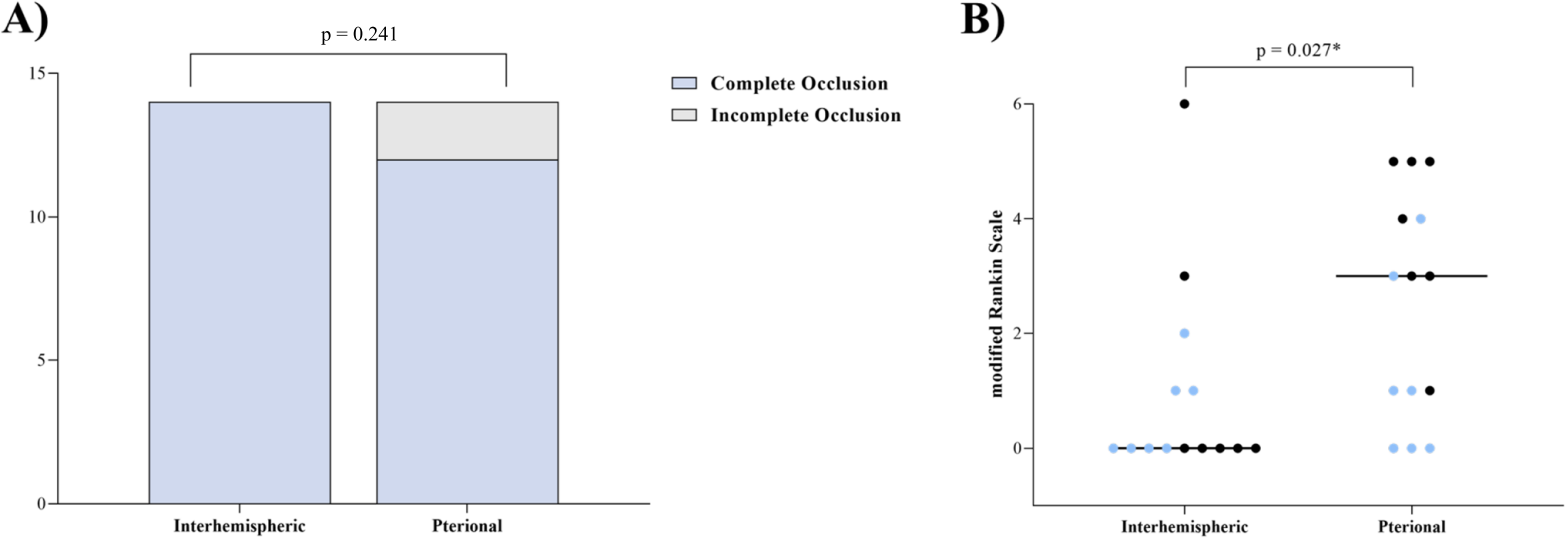
(**A**) the rate of complete occlusion, and (**B**) the dispersion of scores on the mRS among patients with AcomA. Comparative analyses are conducted between two distinct cohorts: one operated using the AIA and a correspondingly matched group treated through the PA

In the analysis of primary outcome parameters, no difference in the degree of occlusion of the investigated AcomA was observed between PA and AIA (*p* = 0.241). However, in terms of the clinical outcome of patients undergoing AcomA surgery, the AIA was associated with a lower mRS at discharge, indicating a better outcome (*p* = 0.027) (Fig. 7).

**Fig. 8:**
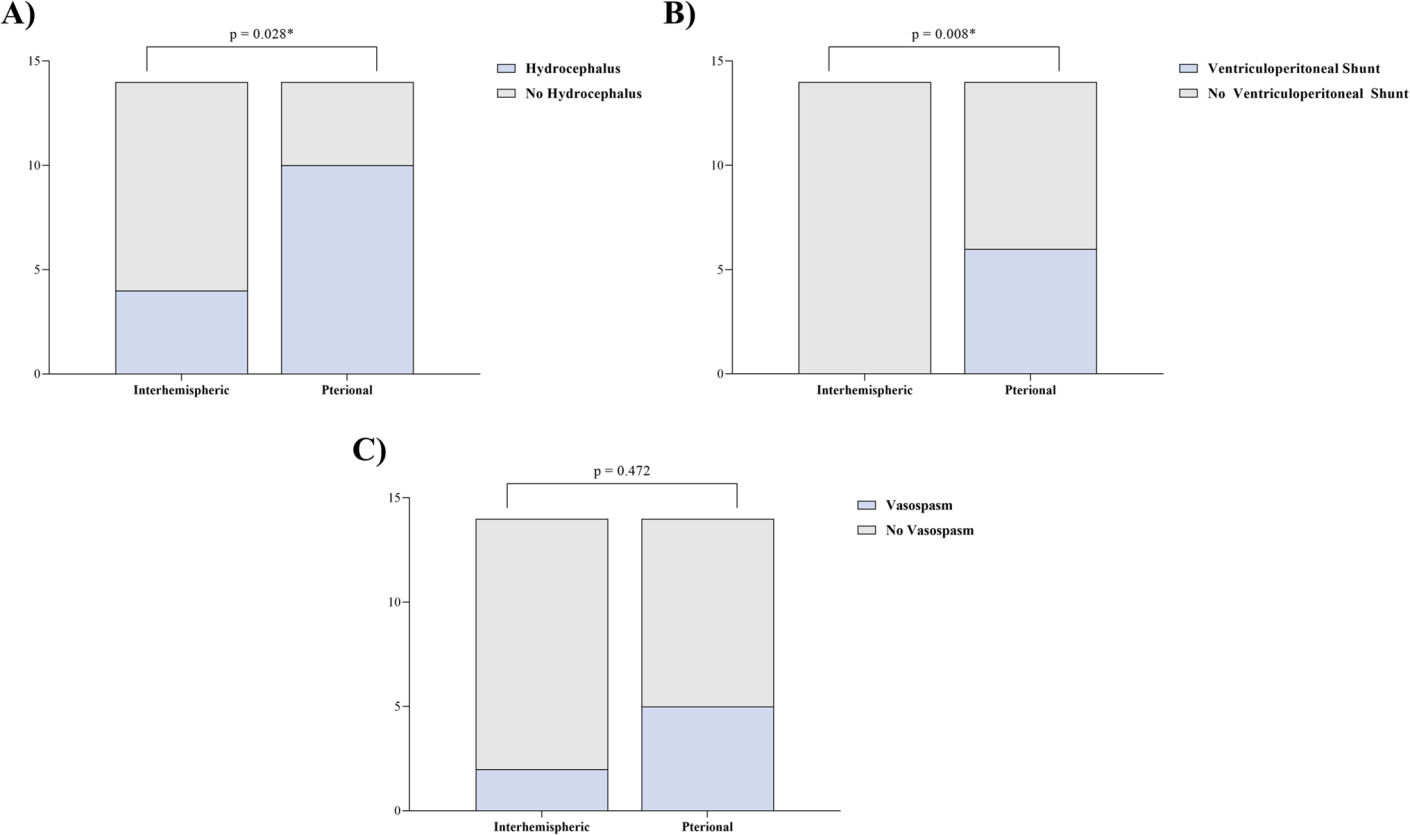
Comparateive evaluations of AcomA managed via AIA and PA, specifically focusing on (**A**) the incidence of postoperative hydrocephalus, (**B**) the consequent need for ventriculoperitoneal shunt implantation, and (**C**) the occurrence rate of vasospasm

Patients undergoing AcomA surgery via the AIA had a significantly lower incidence of hydrocephalus (*p* = 0.028) and required permanent ventriculoperitoneal shunt placement less frequently (*p* = 0.008). The choice of surgical approach did not influence the rate of vasospasms in our cohort (*p* = 0.472) (Fig. 8).

**Fig. 9:**
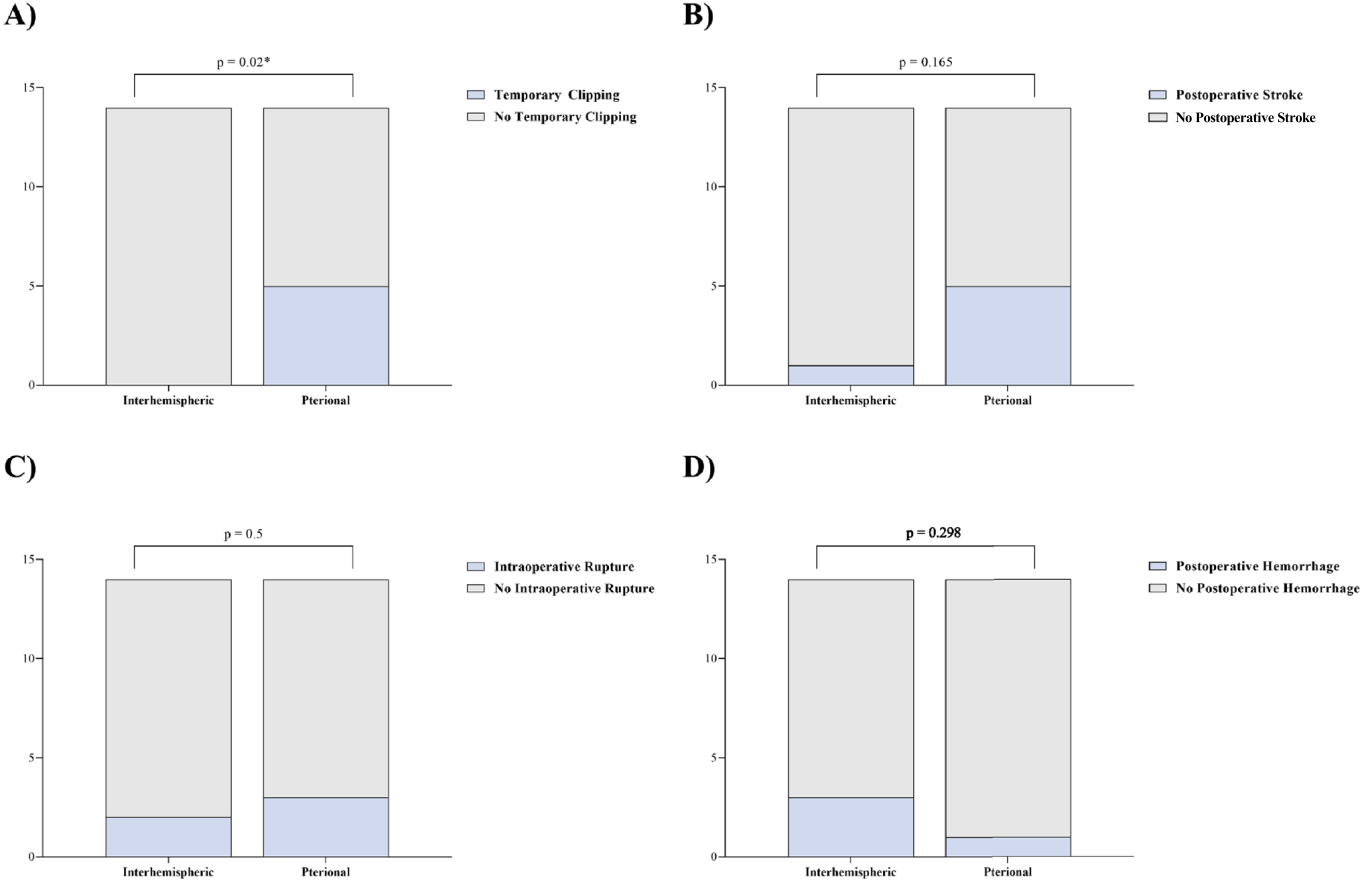
Comparison between AIA and PA to manage AcomA, detailing (**A**) the frequency of temporary clipping and (**B**) its subsequent association with postoperative strokes, along with (**C**) the incidence of intraoperative rupture and (**D**) its correlation with postoperative hemorrhage

Furthermore, it was observed that the AIA resulted in a lower frequency of temporary clipping during AcomA clipping (*p* = 0.02). However, this phenomenon did not translate into an increased number of postoperative infarctions on CT scans (*p* = 0.163). Additionally, there were no significant differences between the two surgical approaches in terms of intraoperative ruptures (*p* = 0.5) and postoperative hemorrhages on CT scans (*p* = 0.298) (Fig. 9).

## Discussion

Our study presents a retrospective analysis comparing the AIA and PA for treating AcomA. We assessed data from patients who underwent one of these surgical procedures and met specific inclusion criteria. The study aimed to determine which approach results in better patient outcomes regarding the degree of IA occlusion and the functional outcome of patients after surgery, defined as primary outcome parameters. In terms of secondary outcome parameters, the incidence of temporary clipping, intraoperative rupture, postoperative occurrences of strokes or hemorrhages and complications like hydrocephalus and vasospasm were evaluated. Ensuring the reliability of comparisons, the study applied an effective matching between these two cohorts making further comparisons robust and credible.

The PA, pioneered by Yaşargil in the 1970s, has become the gold standard for accessing the AcomA complex due to its superior exposure, proximal control, and accessibility [28, 29]. This approach facilitates efficient aneurysm clipping, thereby reducing the likelihood of complications [24, 28, 29]. By adhering to standardized incision protocols, ideal head positioning, and meticulous dissections, surgeons can optimize surgical efficacy and postoperative outcomes. A cardinal step within this method is the dissection of the Sylvian fissure. An expansive opening of this fissure significantly diminishes the risk of premature aneurysm rupture and subsequent hemorrhage during the initial stages of surgery. Nevertheless, this technique is not devoid of challenges. The mandated early retraction of the frontal lobe might unintentionally decompress the aneurysm dome prior to the delineation of certain arterial segments, introducing procedural intricacies. Moreover, the frequent necessity to resect parenchymal structures, such as the gyrus rectus, underscores the procedures intricate demands.

Conversely, the AIA, pioneered by Walter E. Dandy in the 1930s and subsequently optimized by neurosurgeons such as Lougheed and Ito [4, 16, 30], has enjoyed widespread adoption, particularly within the Japanese neurosurgical community. This technique is particularly valuable for addressing midline or deeply located aneurysms, providing unparalleled direct access to the AcomA via dissection through the interhemispheric fissure. A significant merit of this approach lies in its midline orientation, which facilitates equal and extensive visualization of both the A1 and A2 segments and the recurrent artery of Heubner. This method is especially beneficial in scenarios where a midline trajectory is preferable, enabling the effective management of IA without compromising adjacent lateral and basal brain structures [6, 24, 30].

In our limited patient cohort, both surgical methods demonstrated similar effectiveness in achieving IA occlusion, suggesting that the AIA may potentially be a viable alternative to the PA. When evaluating post-surgical functional outcomes through the assessment of the mRS, patient outcome in our cohort suggested a superiority of the AIA. Transitioning to secondary parameters, the AIA demonstrated its advantages, notably in minimizing postoperative complications like hydrocephalus and the ensuing necessity for permanent ventriculoperitoneal shunts, which may explain the better patient outcome observed in our cohort. However, in addressing the complex issue of vasospasms, neither technique proved superior. A significant feature of the AIA was its diminished requirement for temporary clipping. However, the use of temporary clipping in the PA did not result in a higher incidence of postoperative infarctions, underscoring the surgical robustness of both methods. Finally, for critical concerns such as intraoperative ruptures and postoperative hemorrhages, both approaches were comparable, with no significant differences observed. When interpreting these results, it is essential to consider that this study involved a limited cohort of 14 cases for each surgical approach. Therefore, the findings should not be uncritically generalized to other patient cohorts. This study aims to encourage a pathology-specific selection of the approach to the AcomA and to consider alternative methods, given sufficient surgeon experience, alongside the standard approach, which in this case is the PA.

This study has several limitations that should be considered when interpreting the results. First, the surgeries were not performed by the same surgeon, but by multiple well-trained neurosurgeons with extensive experience in aneurysm surgery. Although all surgeons were highly skilled and the operations were conducted at the same hospital, there could still be variations in surgical techniques and decision-making that could affect the outcomes. Second, the sample size was relatively small due to the limited number of cases operated via an AIA. This restricts the generalizability of the results and might not capture all potential complications or nuances associated with the approach. No AcomA in our cohort fell into multiple categories. However, the inability to specifically assess these cases based on our classification represents a limitation of our study.

Despite these limitations, the study provides valuable insights into the comparative efficacy of the two surgical approaches in managing AcomA. Future studies with larger sample sizes and perhaps a single surgeon or a more standardized surgical protocol would help to validate and extend these findings. Nevertheless, the multidimensional matching appears to be an efficient tool for better comparison of different surgical strategies.

## Conclusion

In conclusion, the surgical management of AcomA is complex. Both the AIA and PA present distinct advantages. Our results from a restricted sample indicate promising outcomes with the AIA, as a secure alternative depending on the unique characteristics of each patient and the expertise of the surgeon. Further research is essential, and surgical decisions should be meticulously tailored to the individual nuances of each case.

## Supplementary Information

Below is the link to the electronic supplementary material.

Supplementary file1 (XLSX 16 KB)

## Data Availability

The data supporting the findings of this study are not openly available due to reasons of sensitivity and are available from the corresponding author upon reasonable request.
